# Excision of Tumors of the Breast

**Published:** 1880-11

**Authors:** Chas. C. F. Gay

**Affiliations:** Surgeon to the Buffalo General Hospital


### EXCISION OF TUMORS OF THE BREAST.

BY CHAS. C. F. GAY, M. D., SURGEON TO THE BUFFALO GENERAL HOSPITAL.

### SCIRRHUS OF LEFT BREAST—EXCISION.

MRS. T., aged 62 years, has scirrhus of the breast, involving the axillary glands. Tumor in axilla half the size of a hen's egg. The breast is painful, and the patient is anxious to have the tumor removed, although told that its return is inevitable at no very distant period.

On July 15, 1879, chloroform was given when I removed the entire mammary gland, leaving only just sufficient integumental covering to allow of coaptation of the edges of the wound. The axillary tumor was not removed, but afterwards disappeared.

The edges of the wound were drawn together and secured by silver sutures, ad. plaster, and compress applied wet with carbolized water. The patient convalesced rapidly; one-half the wound healed by first intention. The result as to recurrence is unascertained.

### SCIRRHUS OF THE RIGHT BREAST—EXCISION—RECOVERY.

Mrs. B., aged 45 years, had recently ceased to menstruate, and co-incident with her climacteric period, was the activity in the growth of the tumor. It had been eighteen years growing, but had rapidly increased in size during the past few weeks. The tumor had, to the touch, none of the characteristics of scirrhus, neither was there pain, but the size of it made it very cumbersome, and she had come from an adjoining state in order to have the breast removed. On August 2, 1879, the patient was etherized, when I removed the entire breast. There was but slight hemorrhage. The tumor was found, on examination of its inner surface, to be composed of scirrhus. Its weight was three pounds. Silver sutures were employed, and primary union obtained.

The patient was setting up and walking about in ten days, and in thirteen days was allowed to return to her home.

### RECURRENT CARCINOMA OF BREAST; SIXTH OPERATION—EXCISION—ENUCLEATION OF AXILLARY GLANDS.

Anna Freedholm, aged 50 years, very fleshy, enjoyed good health up to 1878, or until two years ago. She first discovered "yellow water" running from her nipple on April 2, 1878, after which her right breast began to enlarge and become painful. Her breast was excised, and this operation has been followed by four other operations; the last was done at this hospital last winter. The disease has again returned; she suffers very much and has again entered the hospital determined to submit to another operation. I have advised against it, and have called a consultation of the staff, all of whom concur in advising against an operation, except one who favors an immediate operation. The disease now extends up into the axilla, the lymphatic glands of which are involved. The patient choosing another operation, on March 19, 1880, she was etherized, and all the diseased tissue removed, including enucleation of the axillary glands. The wound, or rather wounds—there were three of them—involved almost the entire side and one-half of the front of the chest, two of which were eliptical in form; the larger incisions extended downward obliquely from the axilla, following the course of the fibers of the great pectoral muscle; the smaller incisions at the left of the former extended downwards in the same direction, leaving an isthmus of integumental tissue between them. There was a third wound made by cutting transversely from the larger wound posteriorly. The direction of the incisions of the previous operation was transverse, and as coaptation of the edges of the wound was difficult, incisions were made upon either side in order to relieve the tension of the skin. In this final operation the oblique course of the two anterior wounds rendered coaptation possible by the aid of slight traction.

The edges of the wounds were brought together by silver sutures, supported by adhesive straps. Irrigation with solution of carbolic acid, 1 to 30; the drainage tube, with antiseptic treatment throughout were employed. Pulse and temperature remained nearly normal during the after-treatment.

27th. Removed half the sutures.

30th. Removed all the remaining sutures.

April 10th. Wound nearly closed. There has been absence of menstruation for the past two months. The patient left hospital for her home in Pennsylvania, feeling well and greatly encouraged, and has not since been heard from.

CANCER OF THE RIGHT BREAST—EXCISION—ENUCLEATION OF AXILLARY GLANDS—PRIMARY UNION.

Mrs. W., aged 71 years. Although the tumor had existed for some time, it had only recently become so painful as to make removal necessary. It was not large, but the whole breast, which was small, was involved with enlargement of axillary glands.

April 24th, 1880. She was put under chloroform, assisted by Drs. Ring and Greene, I removed the entire breast by oblique incisions extending from the axilla to a suitable distance below the gland, in the direction of the fibres of the great pectoral muscles. It required considerable time and much care to accomplish the enucleation of the axillary glands which were large; on removal of which the axillary artery could be seen pulsating. There was but little hemorrhage. Wound was closed by silver sutures.

29th. Sutures removed. No suppuration. On May 9th the wound was closed; the pulse and temperature never rising more than one beat and one degree above the normal standard at any time during treatment. Adhesive plaster was at first used, compresses moistened with carbolized water, 1 to 20, applied. The old lady made a good recovery, and when seen several months afterwards, was in the enjoyment of good health, and expressed much gratitude that she was rid of her painful breast.

### CARCINOMA OF LEFT BREAST—ENLARGEMENT OF AXILLARY GLANDS—EXCISION.

Mrs. M. M., aged 49 years, widow, ceased menstruation four years ago. Tumor first appeared two years ago, and she thinks it was caused by a blow upon her breast. The tumor is very large and there is considerable adipose tissue.

April 9th, 1880. Patient was put under ether and the tumor, including the axillary glands, I removed by two curved incisions extending obliquely downward. There was a great deal of hemorrhage, several arteries requiring the ligature.

The wound was brought together by silver sutures, and dressed by adhesive straps and compress. The drainage tube, frequent irrigations, and antiseptic treatment were employed; notwithstanding this treatment there was much suppuration, and on April 26th I have noted that the discharge is offensive, with wound only partially closed. This patient at last made a good recovery.

Her tardy recovery is attributed to the extensive incisions which were necessary in order to include the entire disease.

### ADENOMA OF RIGHT BREAST—ENUCLEATION—PRIMARY UNION.

Miss M., aged 21 years, had concealed from her parents the fact that she had a tumor of her breast, she herself believing it to be cancer, and that she was sure to die of it. The thought of the existence of a cancer preyed so heavily upon her mind that her health became much impaired, and at length the cause of her illness was revealed. So soon as she was assured that the tumor was non-malignant she readily assented to its removal. It occupied a position just above the mammary gland; was the size of the closed hand; nodulated, indeed it consisted of three tumors in juxtaposition. It had been growing for many months.

Oct. 27. Patient was etherized by Dr. Smith, who assisted in the operation of enucleation. Three silver sutures were used and primary union obtained.

*Remarks*—The case of Mrs. F. is especially instructive and valuable since it illustrates how surely and readily an extensive wound will heal, when made upon old cicatricial tissue without causing any rise of pulse or temperature. The case is valuable since it impresses one with the conviction that these tumors should not be abandoned after a second or third operation. It furnishes justification for iterative removal of recurrent carcinoma, and must exert more or less influence upon the mind of any one cognizant of it, when endeavoring to reach a decision as to the propriety of advising an operation. This patient will finally die from the same disease located in the cicatrix or upon some internal viscus, but prolongation of life, and greater immunity from pain with an operation, ought abundantly to compensate one for the hazard of an operation under an anæsthetic, and the discomfort growing out of the after-treatment. It cannot be claimed that these several patients all recovered. Two of them, at least, gave early evidence that the disease would soon return and terminate fatally. It is a singular fact, however, that not a single death of any one of the cases has yet come to my knowledge.

